# Comparative HPTLC study for simultaneous determination of ivabradine and metoprolol using UV and fluorescence detectors

**DOI:** 10.1186/s13065-023-01025-x

**Published:** 2023-09-14

**Authors:** M. Rizk, Shereen Mowaka, Mariam Mohamed, Maha M. Abou El-Alamin

**Affiliations:** 1 Pharmaceutical Analytical Chemistry Department, Faculty of Pharmacy, Helwan University, 11795, Cairo, Egypt; 2 Pharmaceutical Chemistry Department, Faculty of Pharmacy, The British University in Egypt, El-Sherouk City, 11837 Egypt

**Keywords:** Ivabradine, Metoprolol, HPTLC- densitometry, Fluorescence detection mode, UV absorbance detection mode, Green assessment

## Abstract

New, simple, accurate, sensitive and validated high performance thin layer chromatographic (HPTLC) method coupled with UV absorbance mode and fluorescence (FL) detectors which were used for simultaneous determination of ivabradine (IVA) and metoprolol (MET) in their bulk and pharmaceutical dosage form using TLC silica 60 F_254_ plates and non-fluorescent TLC silica gel 60 plates. The developing system was chloroform: methanol: formic acid: ammonia (8.5:1.5:0.2:0.1, v/v). Desnitometric analysis in UV absorbance mode was set at λ = 275 nm. While, fluorescence mode was performed with excitation at 260 nm for quantitative simultaneous determination of IVA and MET using optical filter K320. The retention factors values were 0.45 ± 0.05 and 0.89 ± 0.01 of IVA and MET, respectively. UV absorbance mode, linearity was 50.0–600.0 ng/band for IVA and 50.0–900.0 ng/band for MET. For fluorescence mode, the linearity ranges were 18.0–400.0 ng/band and 50.0–550.0 ng/band for IVA and MET; respectively. ICH guidelines were followed in respect to linearity and range, accuracy, precision and selectivity, limit of detection (LOD), limit of quantitation (LOQ) and robustness. The analytical eco-scale, green analytical procedure index (GAPI) and analytical greenness metric tools were used to assess the suggested method. The quantitative proposed method results showed there was no statistically significant difference at 95% confidence when compared to the reported method of high performance liquid chromatography (HPLC).

## Introduction

**Fig. 1:**
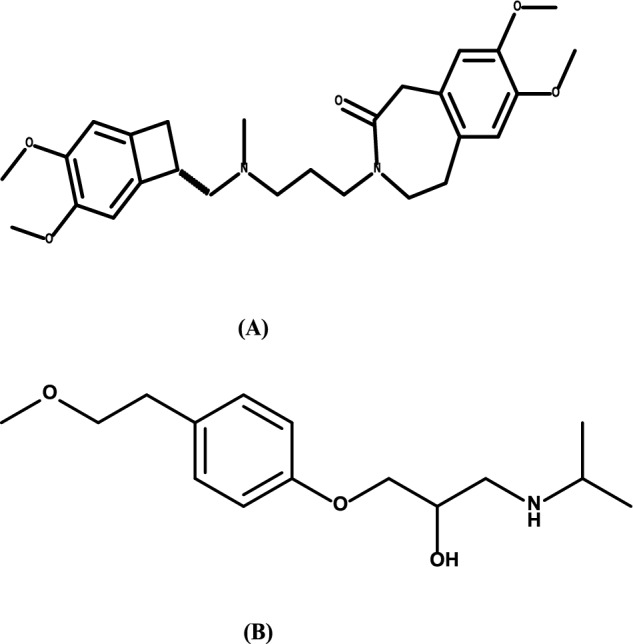
Chemical structures of **A** IVA and **B** MET

Angina is described as chest pain or discomfort that frequently worsens with physical activity or stress and during an episode of angina the patient may experience shortness of breath, fatigue, nausea and dizziness. Myocardial oxygen demand and supply are typically imbalanced that happen in angina. The angina can caused by atherosclerosis, coronary heart disease (CHD), and microvascular dysfunction (MVD) [1]. Ivabradine hydrochloride (IVA), 3-[3-[[(7S)-3,4-dimethoxy-7-bicyclo[4.2.0]octa-1,3,5-trienyl]methyl-methylamino]propyl]-7,8-dimethoxy-2,5-dihydro-1H-3-benzazepin-4-one[2] as shown in (Fig. 1A), is used in treatment of symptomatic management of stable angina pectoris and heart failure. It was approved by the food and drug administration (FDA) on in 2015. Ivabradine acts on blocking hyperpolarization-activated cyclic nucleotide-gated (HCN) channels which responsible for pace maker that regulate heart rate without effecting other cardiac ionic current. Ivabradine selectively inhibits the sodium and potassium ions flow to the channels which lead to slow heart rate. As a result, myocardial oxygen demand is reduced and oxygen supply improves [3–5]. Metoprolol (MET), 1-[4-(2-methoxyethyl)phenoxy]-3-(propan-2-ylamino)propan-2-ol [6]. (As presented in Fig. 1B), is a selective β1 receptor blocker used to treat hypertension, angina pectoris and heart failure [7, 8]. Therefore, the number of angina attacks and heart rate are reduced which lead to improve quality of life. Moreover, the log P and pka were 3.17, 9.37 for IVA and 2.15, 9.7 for MET [9, 10]. In previous literature review, other analytical methods were found for determination of combination ivabradine and metoprolol. These methods involved spectrophotometric method [11], Ultra-high performance chromatography [12, 13], Ultra-high performance chromatography-tandem mass spectrometry [14]. The high performance thin layer chromatography (HPTLC) is used as qualitative and quantitative analysis tool for determination of the drugs. The separation of drugs depend on the nature of adsorbent material that used on plate, physical and chemical properties of analyte and the chosen mobile phase. One thin layer chromatography TLC plate can be used for separation of many samples parallel to each other in one run. Moreover, minimum volume of solvent was used for separation of a mixture with privilege of sensitivity of our proposed HPTLC method, short time of analysis and cost-effectiveness [15, 16]. In the previously reported methods, ivabradine was determined using different TLC system which include aqueous (methanol: water and acetonitrile: water) and non-aqueous (methanol: acetonitrile and methanol: dimethyl sulfoxide) as well as non-aqueous with buffered as binary mobile phases with various concentrations range. Many high performance TLC stationary phase were tired: Kieselgel 60 F_254_ S, Kieselgel 60 CN F_254_ S, Kieselgel 60 NH_2_F_254_ S, RP-8 F_254_ S and RP-18 F_254_ S. Moreover, the association between the log (1/retention factor) values, molecular polarizabilities of solvents and thermodynamic dependence were studied to analyze the effect of the electrostatic interaction to notice if there is influence on retention factor of ivabradine [17, 18]. It is worthy to mention that up till now there is no previously reported HPTLC method for simultaneous determination of ivabradine and metoprolol. Therefore, the aim of the presented work is to develop a quantitative simultaneous determination for ivabradine (IVA) and metoprolol (MET) in their bulk and pharmaceutical dosage using high performance thin layer chromatographic (HPTLC) method using two detectors UV absorbance mode and fluorescence (FL). The three green assessment tools were used to evaluate the suggested method.

## Experimental

### Materials and methods

Ivabradine hydrochloride (IVA) pure sample with 99.90% purity of raw material was donated from the national organization for drug control and research (NODCAR).Metoprolol tartrate (MET) standard was bought from Sigma Aldrich Co., Cairo, Egypt. The purity of MET was provided by company to be 99.90%.Bradipect 5 mg^®^ tablets (Batch No.B08940622) labeled to contain 5 mg ivabradine were bought from local market. Seloken Zoc 25 mg^®^ tablets (Batch No.200277) labeled to contain 25 mg metoprolol tartrate were obtained from local market. Seloken Zoc 50 mg^®^ tablets (Batch No.200025) labeled to contain 50 mg metoprolol tartrate were bought from local market.

HPLC grade chloroform, methanol and formic acid from Fisher Scientific (Loughborough, Leicestershire, UK) were used. Ammonia solution was obtained from E. Merck (Darmstadt, Germany).

### Instrumentation and chromatographic conditions

The chromatographic separation has been achieved on HPTLC silica 60 F_254_ plates (10.0 cm × 10.0 cm, 0.20 mm thickness, E. Merck, Germany). Samples were applied by using CAMAG Linomat 5 auto-sampler with CAMAG microliter syringe (100.0 μL); (CAMAG, Muttenz, Switzerland). Samples were spotted in the form of bands 4 mm width, 10.0 mm from the bottom, 10.0 mm from the edge and 10.0 mm apart using a nitrogen aspirator. The plates were developed using a CAMAG twin trough glass chamber for 10.0 min. The migration distance was 80 mm. The chamber was saturated with mobile phase that consists of chloroform: methanol: formic acid: ammonia (8.5:1.5:0.2:0.1, v/v) at ambient temperature for 30.0 min. Furthermore, the plates were dried at room temperature. A CAMAG TLC scanner 3 with winCATS^®^ software program was used for scanning and densitometric analysis of the development plates. The slit dimension was (3.0 × 0.45 mm) with reflectance measurement and scanning speed at 20.0 mm/s. Two detectors were used. The First one was for detecting the absorbance mode at 275 nm, deuterium lamp was used as a source of radiation for scanning TLC plates. The second one was for fluorescence detection, mercury (Hg) lamp was used for measuring the intensity of emitted light of IVA and MET after excitation at 260 nm using optical filter K320. For using fluorescence detector, the same instrument and conditions were applied except HPTLC plates were non-fluorescence plates by exposing to hydrochloric acid vapor. Elmasonic S 60 H water bath sonicator (Germany) was used for mixing the solutions in pharmaceutical preparation.

### Standard solutions of IVA and MET

Stock solutions of IVA and MET 1.0 mg/mL were prepared separately by weighing 100.0 mg powder of each cited drug, then transferred each one separately into 100.0 mL volumetric flasks. The final volumes were completed with methanol. 10.0 mL of IVA and MET stock solutions were transferred separately into 100.0 mL volumetric flasks. The volume was completed to the mark with methanol to prepare working solutions of IVA and MET with final concentrations of 100.0 μg/mL.

### Construction of calibration graphs and analysis of pure bulk powders

Various volumes from the working solutions of IVA and MET (0.50, 1.0, 1.50, 2.0, 3.0, 4.0, 5.0 and 6.0 μL) of IVA and, (0.50, 1.0, 2.0, 3.0, 5.0, 6.0, 7.0, 8.0 and 9.0 μL) of MET were spotted on TLC plates using CAMAG Linomat 5 auto-sampler with CAMAG microliter syringe (100.0 μL) to give final concentrations (50.0, 100.0, 150.0, 200.0, 250.0, 300.0, 400.0, 500.0 and 600.0 ng per band) of IVA and (50.0, 100.0, 200.0, 250.0, 300.0, 500.0, 600.0, 700.0, 800.0 and 900.0 ng per band) of MET. The HPTLC silica 60 F_254_ plates were developed under prior chromatographic conditions that mentioned in Sect. “Instrumentation and chromatographic conditions”. Densitometric analysis of TLC plates was carried out in absorbance mode at wavelength 275 nm. The calibration curves were constructed by plotting the average peak area against its concentration and regression equations were computed. For fluorescence detection, different volumes of working solutions of IVA and MET (0.18, 0.25, 0.35, 0.50, 0.90, 2.50, 3.0 and 4.0 μL) of IVA and, (0.50, 0.90, 1.0, 2.0, 3.0, 4.0, 5.0 and 5.50 μL) of MET were applied on HPTLC non-fluorescent plates in triplicate to obtain concentration ranges of 18.0–400.0 ng/band of IVA and 50.0–550.0 ng/band of MET. The developed plates were scanned under mentioned Sect. “Instrumentation and chromatographic conditions”. The average peak areas plotted against their corresponding concentration for calibration curves construction. The regression equations and correlation coefficient were computed.

### Laboratory prepared mixtures

Various mixtures including different ratios of IVA and MET were prepared from their working solutions 100.0 μg/mL. Mixtures were spotted on plates and developed under previously mentioned Sect. “Instrumentation and chromatographic conditions”. The peak areas scanned and recorded using both absorbance mode and fluorescence detectors. % Recoveries and standard deviation were calculated.

### Analytical application

IVA and MET (5/25 mg) combined dosage form was not available in the local market. So, synthetic tablets of mixture simulating through combining Bradipect 5 mg^®^ tablets labelled to contain 5 mg of IVA and Seloken Zoc 25 mg^®^ tablets labelled to contain 25 mg of MET. Five tablets of Bradipect and Seloken Zoc were separately accurately weighed and grinded into fine powder. To simulate one tablet, an equivalent weight of 5 mg of IVA and 25 mg of MET were weighed separately from each tablet powder and transferred to 50.0 mL beaker where 50.0 mL of methanol was added. The solution was sonicated for 30.0 min and transferred to 100.0 mL volumetric flask. The volume was completed with methanol. The solution was filtered with 0.22 μm syringe filter. The final sample stock concentration was 50.0 μg/mL of IVA and 250.0 μg/mL of MET. Different volumes of sample stock of IVA and MET (1.80, 2.0 and 3.0 μL) were spotted on silica 60 F_254_ TLC plates to obtain concentrations (90.0, 100.0 and 150.0 ng/ band) of IVA accompanied with (450.0, 500.0 and 750.0 ng/ band) of MET. The plates were scanned using TLC scanner absorbance mode at 275 nm. For fluorescence detection, volumes of sample stock of IVA and MET (1.0, 1.80 and 2.0 μL) were spotted on TLC plates to obtain concentrations (50.0, 90.0 and 100.0 ng/band) of IVA accompanied with (250.0, 450.0 and 500.0 ng/band) of MET. The non-fluorescence plates development and scanning was carried out as mentioned under Sect. “Instrumentation and chromatographic conditions”. The same previous steps were applied for preparing the binary dosage form mixture of IVA and MET (5/50 mg). Bradipect 5 mg^®^ tablets labelled to contain 5 mg of IVA and Seloken Zoc 50 mg^®^ tablets labelled to contain 50 mg of MET which recommended to be co-administrated for antianginal patients. The prepared sample stock concentration was 50.0 μg/mL of IVA and 500.0μg/mL of MET. Accurate volumes (1.20, 1.40 and 1.60 μL) were applied on TLC plates to obtain final concentrations (60.0, 70.0 and 90.0 ng/ band) of IVA with their corresponding (600.0, 700.0 and 900.0 ng/band) of MET. The developed on silica 60 F_254_ TLC plates which UV absorbance mode were scanned at 275 nm. For fluorescence detection, volumes of sample stock of IVA and MET (0.70, 0.80 and 1.0 μL) were spotted on non-fluorescence TLC plates to obtain concentrations (35.0, 40.0 and 50.0 ng/band) of IVA accompanied with (350.0, 400.0 and 500.0 ng/band) of MET. The peak areas of the of corresponding concentrations were scanned using fluorescence detector of TLC scanner. The found concentrations were calculated from their regression equations, respectively. % Recoveries and standard deviation were calculated.

## Results and discussions

The main advantage of HPTLC is the less consumption of the mobile phase and large sample capacity that can be applied on one TLC plate which leads to shorter time of analysis and cost-effectiveness [19]. Moreover, this technique can be applied in the field of pharmaceutical analysis and quality control laboratories [15]. The aim of work is the simultaneous determination of two investigated drugs IVA and MET in their bulk and combined dosage form using UV absorbance mode and fluorescence detectors by using novel, rapid, simple and sensitive HPTLC method where there is no previously reported method for simultaneous determination of both drugs. The sensitivity of the proposed method of using UV and fluorescence detectors was higher than other reported HPTLC methods in the literature review of the two investigated drugs separately as presented in Table 1**.**

**Table 1:** Summary of the reported chromatographic methods for the determination of IVA and MET and our proposed method

Analyte	Matrix	Mobile phase	Stationary phase	Quantitative range of our studied drugs	References
IVA	Dosage form	Chloroform: methanol (1:1 v/v)	Precoated silica gel aluminum plate 60 F _245_	400–2000 ng/band	[25]
IVA		Ethyl acetate: 0.389 M ammonium acetate in methanol (1:5, v/v)		1200–2800 ng/band	[26]
ALMO and MET		Toluene: ethylacetate: methanol: triethylamine(4:1:1:0.4, v/v/v)		3800–13300 ng/spot	[27]
CIL and MET		Toluene: chloroform: methanol: glacial acetic acid (45:25:25:5, v/v/v)		500–2500 ng/spot	[28]
ALMO and MET		Methanol: ethylacetate: water: toluene: 25% ammonia (1.5:5.0:0.3:0.3, v/v)		180–280 μg/mL	[29]
SMIN and MET		Methanol: Ethylacetate:trimethylamine (6:4:0.1, v/v/v)		1000–7000 ng/band	[30]
HCTZ and MET	Plasma	Chloroform: methanol:ammonia (9:1:0.5 v/v/v)		2000–12000 ng/band	[31]
IVA and MET	Dosage form	Orthphosphoric acid (0.1%) buffer: acetonitrile (60:40 v/v)	Denali C_18_ reverse-phase RP-HPLC	• 5–30 μg/mL for IVA • 25–150 μg/mL for MET	[13]
IVA and MET		Chloroform: methanol: formic acid: ammonia (8.50:1.50:0.20:0.10, v/v)	Precoated silica gel aluminum plate 60 F _245_	For UV detector • 50.0–600.0 ng/band for IVA • 50.0–900.0 ng/band for MET For fluorescence detector • 18.0–400.0 ng/band for IVA • 50.0–550.0 ng/band for MET	Our proposed method

*ALMO* amlodipine, *CIL* cilnidipine, *HCTZ* hydrochlorothiazide and *SMIN* isosorbide mononitrate

### Method optimization for the HPTLC-densitometric measurements

The consecutive parameters need to be optimized in order to get good separation, symmetric sharp peaks, and acceptable retention factors for the cited drugs.

#### Mobile phase

**Fig. 2:**
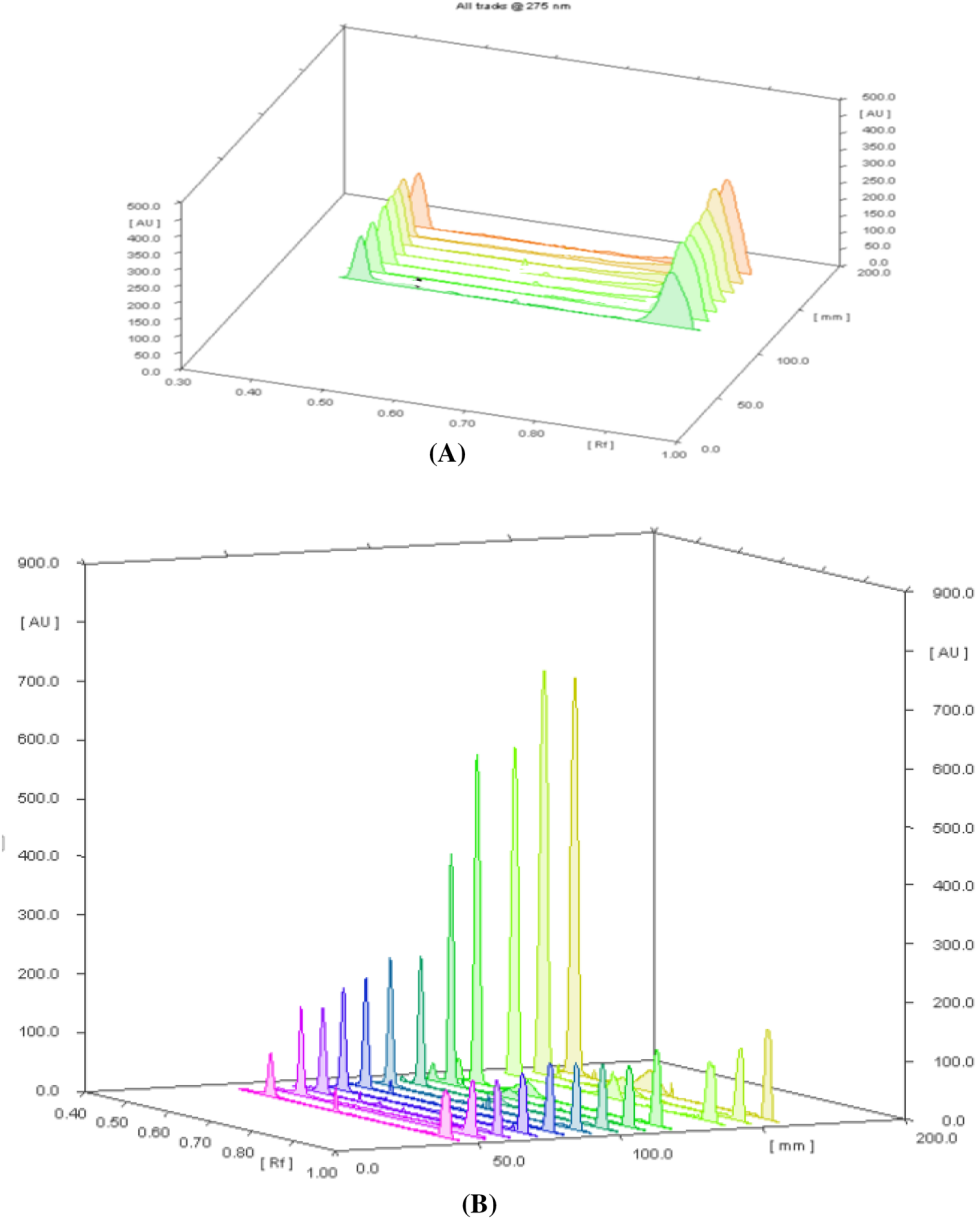
3D densitograms of IVA and MET using **A** Abs detection mode and **B** fluorescence detection mode

Several trials of mobile phases have been applied that composed of dichloromethane: methanol (9:1, *v/v*), toluene: ethyl acetate: methanol: trimethylamine (8: 1:1:0.4, *v/v*), toluene: propanol: methanol: trimethylamine (8:1:1:0.5, *v/v*), ethyl acetate: methanol (9:1, *v/v*) and chloroform: methanol: ethyl acetate: formic acid (7.5: 2: 0.5: 0.01, *v/v*). The optimum mobile phase system composition showing the best resolution and separation of two cited drugs was chloroform: methanol: formic acid: ammonia (8.5:1.5:0.2:0.1, *v/v*). Formic acid was used to reduce peak tailing of the drugs. The R_f_ values were 0.45 ± 0.05 and 0.89 ± 0.01 for IVA and MET, respectively as shown in Fig. 2**.**

#### Stationary phase

For UV detection at 275 nm, TLC pre-coated silica gel aluminum plates 60 F_254_ were used for simultaneous determination of IVA and MET in bulk and pharmaceutical dosage form. For fluorescence reflection mode detector at lambda excitation wavelength λex of 260 nm and K320 as cut off, the TLC silica gel plates 60 F_254_ were exposed to hydrochloric acid vapors for few minutes in a closed chamber which break down the fluorescent material on TLC [20]. Consequently, the plates were converted into non-fluorescent plate (TLC silica gel 60) after air drying for an hour. The non-fluorescence plates were used for fluorescence detection. This step was important to be done because the peaks of the cited drugs appear inform of inverted peaks.

#### Optimum wavelength selection

For UV detection, different wavelengths were tried as 230, 260, 275, 285 and 290 nm. 275 nm was selected for determination of IVA and MET as the chosen excitation wavelength gave good resolution and sensitivity. For fluorescence mode detection, various excitation wavelengths (260, 270, 275 and 285 nm) and cut off filters (K320 and K400) were tested. But, all of them gave bad resolution and asymmetric peaks. The optimum excitation wavelength was 260 nm with using K320 cut off filter which gave sharp symmetric peaks and high fluorescence intensity for quantitative determination of IVA and MET.

#### Slit dimensions of scanning light beam

The optimum slit dimensions of scanning light beam of bands on TLC plates were (3.0 × 0.45 mm) which gave maximum sensitivity of the two cited drugs and good selectivity.

#### Time of saturation

The same degree of vapor saturation should be used each time where the plate is developed in order to get reproducible results [15]. Several saturation times from 10 to 40 min were tested to select the most proper time. The good results were obtained starting from 25 min for saturation time; consequently, a time of 30 min was selected as the appropriate saturation time.

#### Migration distance and development time

To prevent the mobile phase evaporation from the plate edges and achieve a uniform movement of the analytes in the stationary phase [15], 80 mm was chosen from the beginning for optimum migration distance in order to attain the best resolution. The distance of migration was carried out within 10.0 min.

### Method validation

The proposed method had been validated according to international conference on harmonization ICH guidelines regarding to linearity and range, accuracy, precision, selectivity, limit of detection (LOD) and limit of quantitation (LOQ) and robustness [21]. The results are displayed in Table 2**.**

**Table 2:** Analytical parameters of proposed method for simultaneous determination of IVA and MET by applying HPTLC method using absorbance (Abs) and fluorescence (FL) detectors

Detection mode	HPTLC- Abs		HPTLC –FL	
Drugs	IVA	MET	IVA	MET
Parameters				
Concentration range (ng/band)	50.0–600.0	50.0–900.0	18.0–400.0	50.0–550.0
Correlation coefficient (R^2^)	0.9994	0.9998	0.9999	0.9995
S.D^a^	0.11	0.03	0.21	0.12
% RSD^b^	0.11	0.03	0.21	0.12
Slope	5.2116	13.084	3.5199	1.2348
Intercept	594.61	1362.5	519.12	1468.2
Standard deviation of intercept	25.93	65.96	6.28	6.19
Limit of detection (LOD), (ng/band)	16.42	16.63	5.89	16.56
Limit of quantitation (LOQ), (ng/band)	50.0	50.0	18.0	50.0

Acceptance criteria of ^a^S.D: Standard deviation and ^b^% RSD: Relative standard deviation values were less than 2 according to ICH guidelines

#### Linearity and range

A linear relationship was obtained between peak areas and their corresponding concentrations in range of (50.0–600.0 ng/band) and (50.0–900.0 ng/band) for IVA and MET, respectively. After the development of TLC silica gel plates 60 F254 under the previously mentioned chromatographic conditions and scanned using UV absorbance mode. The linearity was (18.0–400.0 ng/band) and (50.0–550.0 ng/band) for IVA and MET after using fluorescence mode, respectively, as presented in Table 2**.**

#### Accuracy

For absorbance mode, (150.0, 400.0 and 600.0 ng/band) of IVA and (250.0, 300.0 and 800.0 ng/band) of MET were chosen to assess the accuracy of the proposed method. For fluorescence mode, (30.0, 100.0 and 400.0 ng/band) of IVA and (90.0, 300.0 and 500.0 ng/band) of MET were selected to test the accuracy of the proposed method. % Recoveries of three levels of chosen standard solutions of IVA and MET in triplicates were calculated. The results are represented in Table 3**.**

**Table 3:** Accuracy of proposed method for determination IVA and MET by applying HPTLC method using absorbance (Abs) and fluorescence (FL) detectors

HPTLC-Abs				HPTLC-FL			
IVA		MET		IVA		MET	
Conc (ng/band)	% Found^a^	Conc (ng/band)	% Found^a^	Conc (ng/band)	% Found^a^	Conc (ng/band)	% Found^a^
150.0	98.55	250.0	99.80	30.0	99.32	90.0	100.78
400.0	100.95	300.0	99.55	100.0	99.06	300.0	98.21
600.0	98.19	800.0	99.53	400.0	100.56	500.0	98.12
Mean ± SD^b^	99.23 ± 1.50	Mean ± SD^b^	99.63 ± 0.15	Mean ± SD^b^	99.65 ± 0.80	Mean ± SD^b^	99.04 ± 0.51

The % recoveries of bulk were found to be between 98 and 102% and for standard deviation values were less than 2 according to ICH guidelines

^a^Average of three determinations

^b^Standard deviation

#### Precision

Intraday and interday precision were assessed. The intraday precision was tested through the determination of three concentrations level of the two cited drugs on the same day. The interday precision examined by analyzing three concentrations level on the three consecutive days. The calculated % RSD Values were less than 2 which indicate the proposed method has a good precision. As shown in Table 4.

**Table 4:** Intraday and interday precision of proposed method for determination IVA and MET by applying HPTLC method using absorbance (Abs) and fluorescence (FL) detectors

Drug	IVA	HPTLC-Abs	HPTLC-Abs (IVA)
Conc (ng/band)	Intraday precision (% Found^a^ ± % RSD^b^)	Conc (ng/band)	Interday precision (% Found^a^ ± % RSD^b^)
100.0	99.00 ± 0.18	150.0	100.97 ± 0.14
400.0	101.08 ± 0.07	300.0	100.0 ± 0.09
600.0	98.89 ± 0.05	500.0	99.70 ± 0.06

Drug	MET	HPTLC-Abs	HPTLC-Abs (MET)
Conc (ng/band)	Intraday precision (% Found^a^ ± % RSD^b^)	Conc (ng/band)	Interday precision (% Found^a^ ± % RSD^b^)
200.0	100.82 ± 0.04	100.0	100.00 ± 0.05
500.0	100.20 ± 0.02	600.0	101.00 ± 0.02
800.0	99.0 ± 0.01	800.0	99.90 ± 0.01

Drug	IVA	HPTLC-FL	HPTLC-FL (IVA)
Conc (ng/band)	Intraday precision (% Found^a^ ± % RSD^b^)	Conc (ng/band)	Interday precision (% Found^a^ ± % RSD^b^)
25.0	100.0 ± 0.32	35.0	98.00 ± 0.31
90.0	100.11 ± 0.21	50.0	100.00 ± 0.25
40.0	99.65 ± 0.10	300.0	101.05 ± 0.08

Drug	MET	HPTLC-FL	HPTLC-FL (MET)
Conc (ng/band)	Intraday precision (% Found^a^ ± % RSD^b^)	Conc (ng/band)	Interday precision (% Found^a^ ± % RSD^b^)
100.0	100.00 ± 0.12	60.0	100.06 ± 0.12
200.0	101.50 ± 0.11	300.0	100.37 ± 0.11
550.0	100.63 ± 0.09	500.0	100.84 ± 0.08

Acceptance criteria of % found of bulk were between 98 and 102% and % Relative standard deviation values were less than 2 according to method validation

^a^Average of three determinations

^b^Relative standard deviation

#### Selectivity

**Fig. 3:**
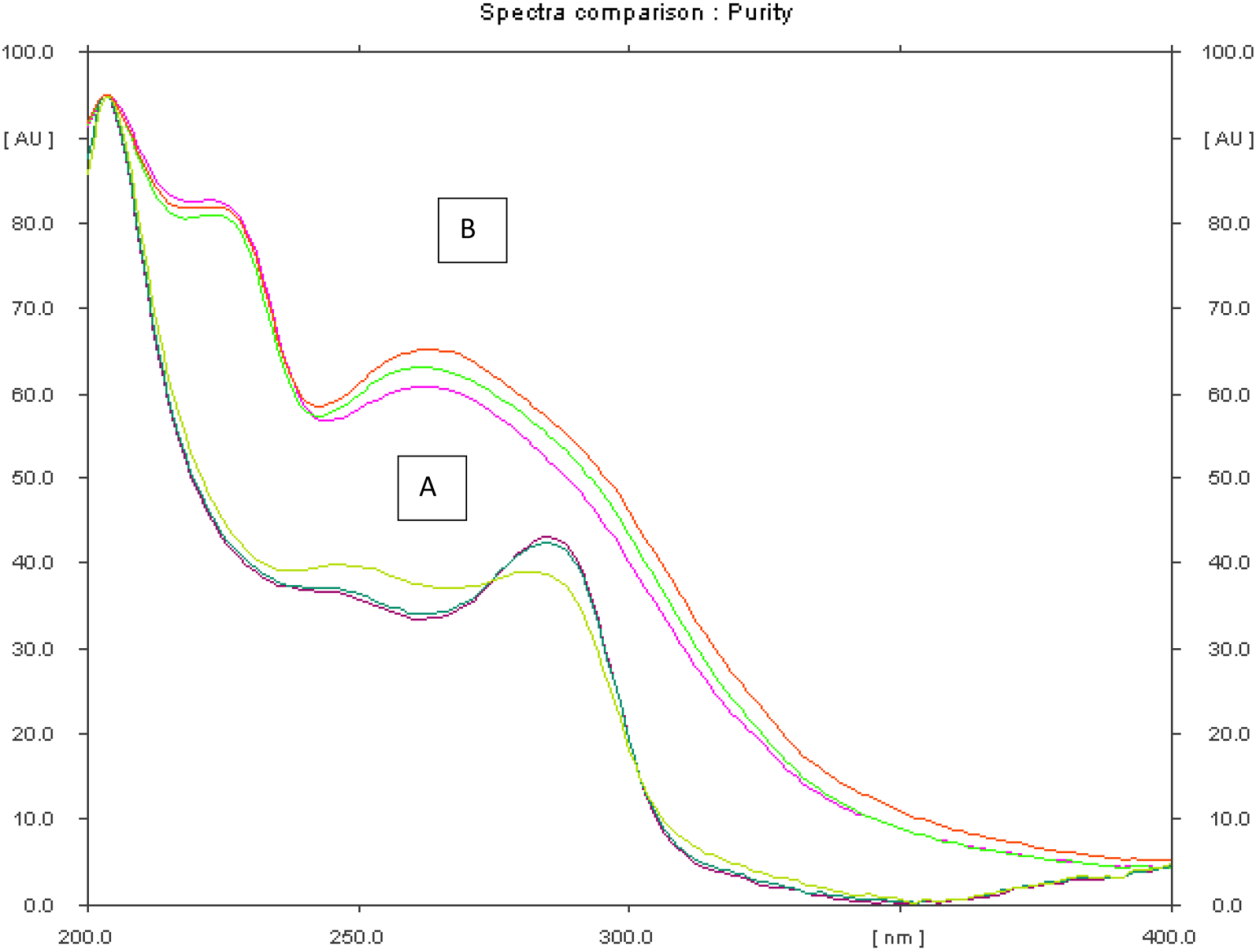
Overlaid spectra of **A** IVA and **B** MET

**Fig. 4:**
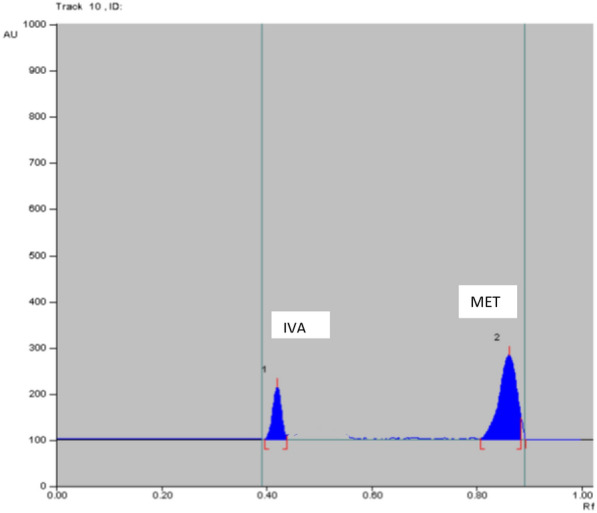
2D HPTLC-UV densitogram of laboratory prepared mixture of 50.0 ng/band of IVA and 500.0 ng/band of MET

**Fig. 5:**
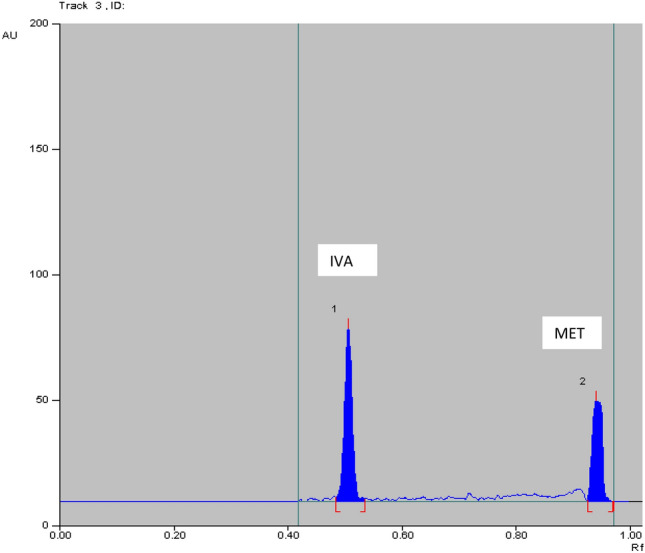
2D HPTLC-fluorescence densitogram of laboratory prepared mixture of 35.0 ng/band of IVA and 500.0 ng/band of MET

The peak purity of IVA and MET were tested. The developed plate was scanned through TLC scanner and absorption spectrum was recorded at different points throughout each peak. The correlation coefficient between the spectra recorded at peak start and peak maximum was 0.9998. The peak maximum and peak end spectra's correlation coefficient was 0.9997 which attained high values. The purity of peaks was obtained from the mathematic analysis of the software as shown in Fig. 3. Selectivity was tested by analyzing laboratory prepared mixtures with different amounts of IVA and MET in several ratios. % recoveries and standard deviations were calculated as shown in Table 5**, **Figs. 4 and 5.

**Table 5:** Determination IVA and MET in laboratory prepared mixtures by applying TLC method using absorbance (Abs) and fluorescence (FL) detectors

Analyte conc. in laboratory prepared mix (ng/band) TLC-Abs		%Recovery TLC-Abs		Analyte conc. in laboratory prepared mix (μg/band) TLC-FL		%Recovery TLC-FL	
IVA	MET	IVA	MET	IVA	MET	IVA	MET
100.0^a^	500.0^a^	100.81	99.91	35.0	500.0	98.11	100.38
50.0	500.0	98.00	99.93	50.0	400.0	98.23	99.57
400.0	700.0	100.99	99.76	100.0^a^	500.0^a^	99.40	100.38
%Recovery Mean ± SD		99.93 ± 1.67	99.87 ± 0.09	%Recovery Mean ± SD		98.58 ± 0.70	100.11 ± 0.47

^a^Data represent the ratio of two cited drugs concentrations (conc.) which found in the pharmaceutical dosage form

Acceptance criteria of % recoveries of bulk were between 98 and102% and standard deviation values were less than 2 according to ICH guidelines

#### Limit of detection (LOD) and limit of quantitation (LOQ)

LOD is the lowest concentration can be detected. However, LOQ is the lowest concentration can be accurately quantified. They are computed according the following equations. LOD = 3.3 σ/S and LOQ = 10 σ /S. Where, σ is the calculated standard deviation of y-intercept and S is the slope from the attained calibration curves. The results are shown in Table 2.

#### Robustness

Robustness was examined by exhibiting that the system is unaffected after deliberately variations of some chromatographic conditions. Some variations were applied as saturation time (± 5 min) and migration distance (± 5 mm). The peak under area of each experimental parameter was used in calculation of the percentage recoveries and relative standard deviations (%RSD). The % RSD values were less than 2 which indicates the good robustness of the proposed method. The results are reported in Table 6.

**Table 6:** Robustness of the suggested method determination of IVA and MET in laboratory prepared mixtures by applying HPTLC method using absorbance and fluorescence detectors

Parameter	% Recovery ± SD^a^ (%RSD)^b^ HPTLC-Abs		% Recovery ± SD^a^ (%RSD)^b^ HP TLC-FL	
Drug	IVA	MET	IVA	MET
Concentration (ng/band)	150.0	750.0	35.0	350.0
Migration distance (8.5 cm)	100.63 ± 0.57 (0.04)	99.03 ± 1.60 (0.01)	98.93 ± 1.73 (0.27)	99.98 ± 0.57 (0.03)
Migration distance (7.5 cm)	100.61 ± 0.28 (0.02)	99.02 ± 1.52 (0.01)	99.47 ± 1.52 (0.23)	100.14 ± 1.00 (0.05)
Saturation time (35.0 min)	100.62 ± 0.40 (0.03)	99.02 ± 1.52 (0.01)	99.06 ± 1.60 (0.25)	100.10 ± 1.44 (0.07)
Saturation time (25.0 min)	100.65 ± 0.86 (0.06)	99.03 ± 1.25 (0.01)	99.47 ± 1.52 (0.23)	100.18 ± 1.60 (0.08)

Acceptance criteria of % recoveries of bulk were between 98 and 102% and standard deviation and % relative standard deviation values were less than 2 according to ICH guidelines

^a^Standard deviation

^b^Relative standard deviation

### Application of the proposed method

**Fig. 6:**
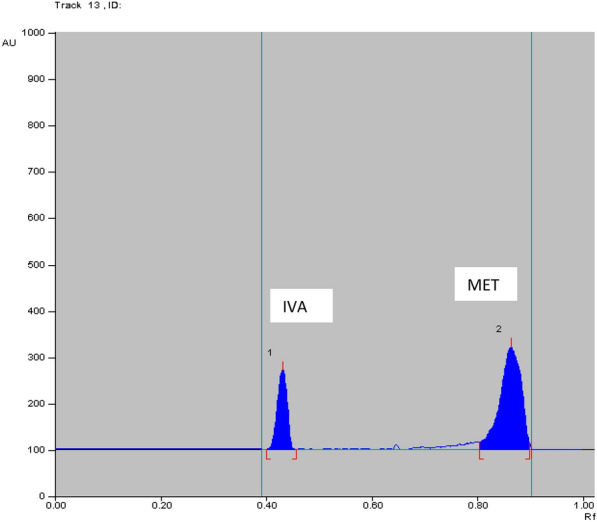
2D HPTLC-UV densitogram of dosage form (5 mg IVA/25 mg MET) of 90.0 ng/band of IVA and 450.0 ng/band of MET

**Fig. 7:**
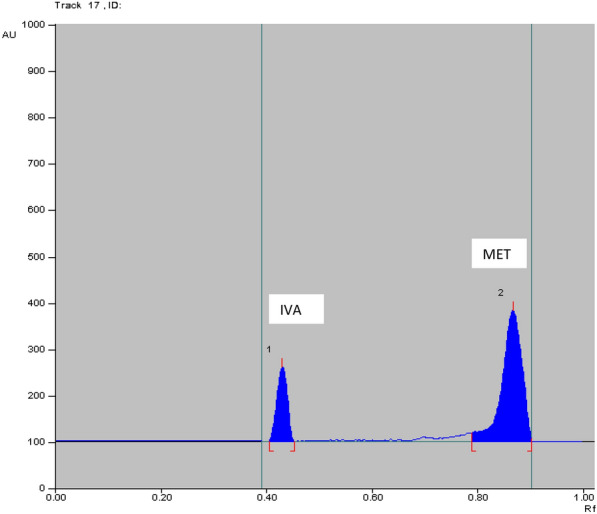
2D HPTLC-UV densitogram of dosage form (5 mg IVA/50 mg MET) of 90.0 ng/band of IVA and 900.0 ng/band of MET

**Fig. 8:**
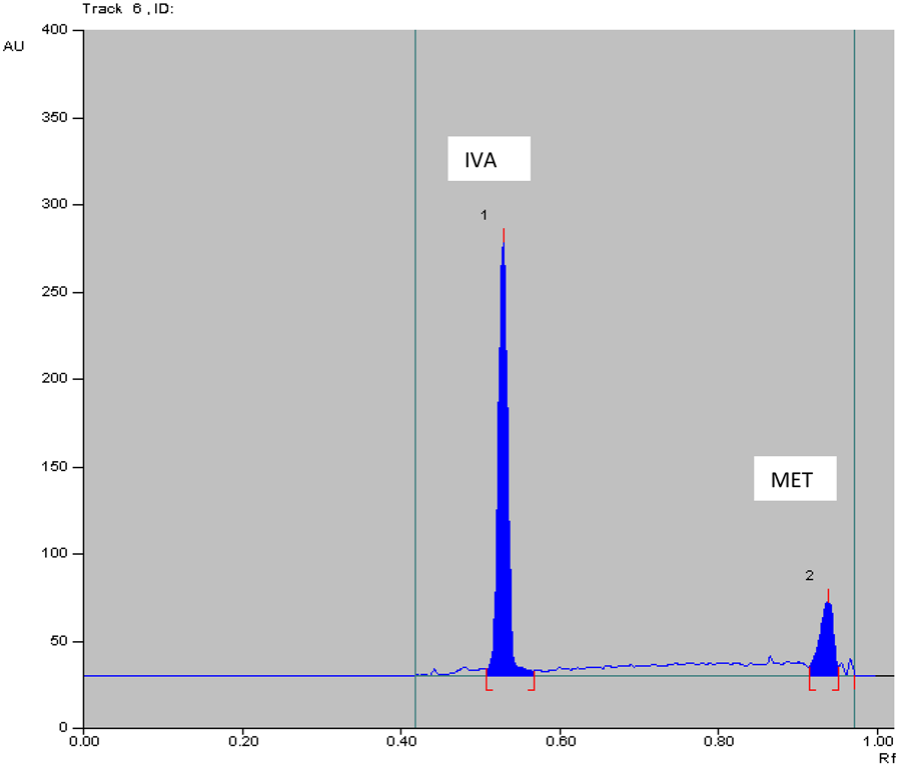
2D HPTLC-fluorescence densitogram of dosage form (5 mg IVA/25 mg MET) of 90.0 ng/band of IVA and 450.0 ng/band of MET

**Fig. 9:**
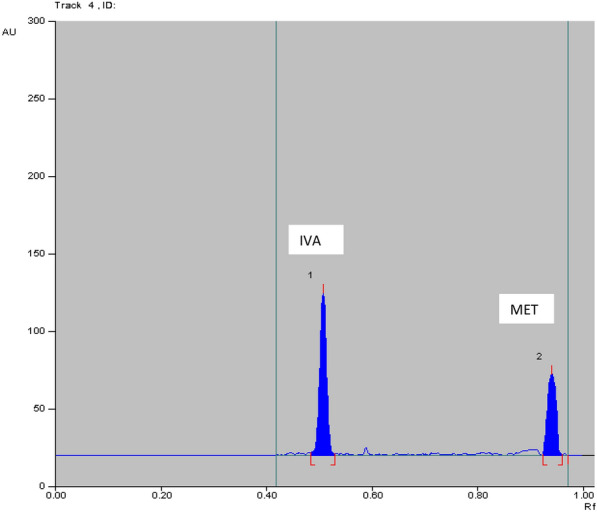
2D HPTLC-fluorescence densitogram of dosage form (5 mg IVA/50 mg MET) of 40.0 ng/band of IVA and 400.0 ng/band of MET

The method was applied for determination of IVA and MET in the simulated simulated combined dosage form. The results are shown in Table 7**.** The satisfactory percentage recoveries were proved that these method can be used for the routine analysis in determination of two studied drugs. As represented in Figs. 6, 7, 8 and 9**.**

**Table7:** Determination of IVA and MET in their simulated combined dosage form by applying HPTLC method using absorbance (Abs) and fluorescence (FL) detectors

Dosage form (ng/band)		Recovery% HPTLC-Abs		Dosage form (ng/band)		Recovery% HPTLC-FL	
IVA	MET	IVA	MET	IVA	MET	IVA	MET
90.0^a^	450.0^a^	100.28	99.14	50.0^a^	250.0^a^	99.93	100.35
100.0^a^	500.0^a^	100.81	99.91	90.0^a^	450.0^a^	98.76	99.30
150.0^a^	750.0^a^	100.59	99.02	100.0^a^	500.0^a^	99.68	100.22
Mean ± SD		100.56 ± 0.26	99.36 ± 0.48	Mean ± SD		99.46 ± 0.61	99.96 ± 0.57
60.0^b^	600.0^b^	100.86	99.83	35.0^b^	350.0^b^	98.11	99.91
70.0^b^	700.0^b^	100.15	99.76	40.0^b^	400.0^b^	98.28	99.57
90.0^b^	900.0^b^	100.28	98.82	50.0^b^	500.0^b^	98.23	100.38
Mean ± SD		100.43 ± 0.37	99.47 ± 0.56	Mean ± SD		98.21 ± 0.08	99.95 ± 0.41

Acceptance criteria of % recoveries of pharmaceutical preparation were between 90 and 110% and standard deviation and % relative standard deviation values were less than 2 according to ICH guidelines

^a^Ratio of 5 mg IVA: 25 mg of MET

^b^Ratio of 5 mg IVA: 50 mg of MET

## Statistical analysis

Table 8 represents the statistical analysis between the proposed method and reported HPLC methods [13]. The calculated t and f values were shown that there was no significant difference between suggested and reported methods.

**Table 8:** Statistical comparison between the results obtained by the suggested method and the reported HPLC method for the determination of IVA and MET in bulk by applying HPTLC method using absorbance (Abs) and fluorescence (FL) detectors

Item	Ivabradine		Metoprolol		Ivabradine		Metoprolol	
	Proposed HPTLC-Abs method	Reported HPLC method [13]	Proposed HPTLC-Abs method	Reported HPLC method [13]	Proposed HPTLC-FL method	Reported HPLC Method [13]	Proposed HPTLC-FL method	Reported HPLC method [13]
Mean^a^ ± SD	99.23% ± 1.50	99.94% ± 0.45	99.63% ± 0.15	99.55% ± 0.57	99.65% ± 0.80	99.94% ± 0.45	99.04% ± 1.51	99.55% ± 0.57
n	3	3	3	3	3	3	3	3
Student’s t-test (2.766)^b^	− 1.57		0.47		− 1.09		− 1.09	
F-value (19.00)^b^	11.11		14.44		3.16		7.02	

^a^Average of three determinations

^b^ Tabulated t and F values at P = 0.05 [32]

## Assessment of greenness of the method: the analytical Eco-scale, green analytical procedure index (GAPI) and analytical greenness metric (AGREE)

**Fig. 10:**
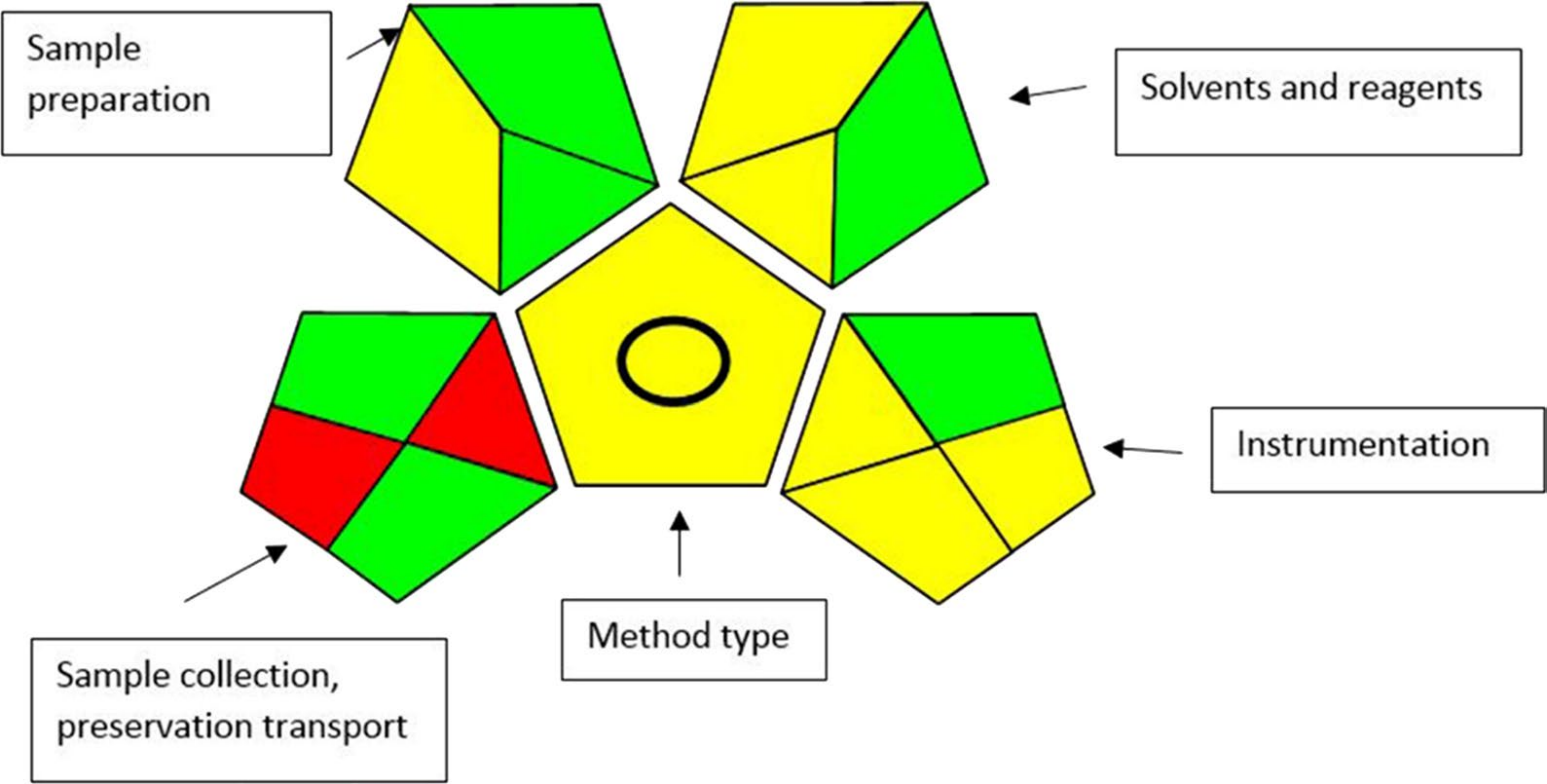
GAPI assessment profile of HPTLC method

**Fig. 11:**
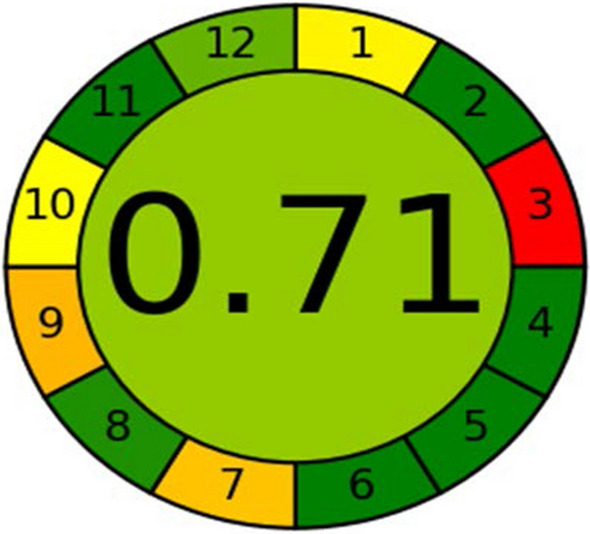
AGREE assessment profile of HPTLC method

The suggested methods were assessed by using three greenness tools. First one, the analytical eco-scale was used for determination the greenness of the method by calculating analytical eco-scale total score; more than 75% which corresponding to excellent green analysis, more than 50% representing acceptable green analysis and less than 50% that meaning inadequate green analysis, as displayed in Table 9 [22]. Second one, green analytical procedure index (GAPI) was evaluated the proposed method by representing five pentagrams of sample collection, sample preparation, reagents and solvents, instrumentation and general method type. Each pictogram has been given one of the three colors; green color that representing low impact on the environment, yellow color that showing medium impact on the environment and red color that indicating high impact on the environment. As shown in Fig. 10**,** sample collection and transport in the first pentagram have red color. As a result of distancing between production and quality control department in the pharmaceutical industry [23].The suggested method represents 6 green regions, 7 yellow regions and red regions. Finally, the analytical greenness metric displayed 12 principles which include sample treatment, amount of sample, device positioning, sample preparation stage, automation and miniaturization, derivatization, waste, analysis throughout, energy consumption, source of reagents, toxicity and operator’s safety to assess the suggested method. Each variable can be given different weights which represent the width of each segment and permit the flexibility in assessing the method. Each principle of green analytical chemistry was converted into a scale 0–1. Moreover, according to the procedure’s performance the red, yellow and green colors scale were expressed to each principle. Figure 11 was shown a graph of the clock with the total score and color in the middle that representing of the proposed method. When the dark green color and overall score close to one are shown in the middle of clock pictogram which indicates that the method is eco-friendly to the environment [24].

**Table 9:** The penalty points used to calculate the eco-scale of our proposed method

Type of reagent	Penalty points
Chloroform	(less than 10 mL) = 4
Methanol	(less than 10 mL) = 6
Formic acid	(less than10 mL) = 6
Ammonia	(less than10 mL) = 6
Hazardousness	(None) = 0
Energy consumption	(less than or equal to 1.5 k Wh per sample) = 1
Waste production	(10 mL) = 3
Total penalty points	26
Analytical eco-scale total score	74
	Acceptable green analysis

## Conclusion

A new, rapid and sensitive suggested HPTLC method coupled with UV absorbance mode and fluorescence detectors were used for simultaneous estimation of IVA and MET in bulk and pharmaceutical preparations. The suggested method revealed advantage over the reported HPLC method as discussed in statistical analysis. The developed method has been validated as stated in ICH guidelines. The analytical Eco-scale, green analytical procedure index (GAPI) and analytical greenness metric were applied to evaluate our suggested method. The method is considered simple and do not need sophisticated sample treatment. The studied method could be routinely used for quantitative determination of the two cited drugs in quality control laboratories.

## Data Availability

The corresponding author will provide the data that supporting the suggested method of this study upon reasonable request.

## Abbreviations

HPTLC: High performance thin layer chromatography
FL: Fluorescence
Abs: Absorbance
IVA: Ivabradine
MET: Metoprolol
TLC: Thin layer chromatography
ICH: International conference on harmonization
HPLC: High performance liquid chromatography
CHD: Coronary heart disease
MVD: Microvascular dysfunction
HCN: Hyperpolarization-activated cyclic nucleotide-gated channels
FDA: Food and drug administration
NODCAR: National organization for drug control and research
λex: Lambda excitation wavelength
Hg: Mercury lamp
R_f_: Retention factor
LOD: Limit of detection
LOQ: Limit of quantitation
%RSD: Relative standard deviations
S.D: Standard deviation
AGREE: Analytical greenness metric
GAPI: Green analytical procedure index
ALMO: Amlodipine
CIL: Cilnidipine
HCTZ: Hydrochlorothiazide
SMIN: Isosorbide mononitrate
Conc: Concentration
